# Dynamic Observation of Lung Nodules on Chest CT Before Diagnosis of Early Lung Cancer

**DOI:** 10.3389/fonc.2022.713881

**Published:** 2022-03-09

**Authors:** Qiaodan Du, Jia Peng, Xiuyu Wang, MingFang Ji, Yuting Liao, Binghang Tang

**Affiliations:** ^1^ Medical Imaging Center, Zhongshan People’s Hospital, Zhongshan, China; ^2^ Cancer Research Institute, Zhongshan People’s Hospital, Zhongshan, China; ^3^ GE Healthcare Pharmaceutical Diagnostics, Shanghai, China

**Keywords:** lung nodules, chest CT, dynamic observation, lung cancer, early diagnosis

## Abstract

**Objective:**

Early recognition and diagnosis of lung cancer can help improve the prognosis of patients. However, early imaging patterns of malignant lung nodules are not fully clear. To understand the early imaging signs of malignant lung cancer nodules, the changes of the lung nodules before diagnosis were dynamically observed and analyzed.

**Materials and Methods:**

This retrospective study observed dynamic changes of lung nodules before pathological confirmation with consecutive regular chest CT examination from January 2003 to December 2018. At least 3 follow-up CT scans were performed in all cases, and the interval between each follow-up was about 1 year. The size, density, and morphological signs of the nodules were evaluated based on the CT axial image, and a reverse line chart or scatter plot with the diagnosis time as coordinate origin was constructed.

**Results:**

A total of 55 lung nodules in 53 patients (mean age, 58.40 years ±11.43 [standard deviation]; 20 women) were accessed. The follow-up time was 5.96 ± 2.68 years. The average diameters in maximum slice of the lesion at baseline and last scan were 6.83 ± 2.92 mm and 16.65 ± 7.34 mm, respectively. According to the reverse line chart, the nodule growth curve segments within 4 years from the last scan showed an ascending shape, and those beyond 4 years showed a flat shape. There are 90.9% (50/55) GGN and 9.1% (5/55) SN when the lesion first appears, and 21.8% (12/55) GGN, 38.2% (21/55) PSN, and 40% (22/55) SN in the last scan. There are 12.7% (7/55) and 98.2% (54/55) nodules with poor morphological signs at baseline and last scan, respectively.

**Conclusion:**

At the time node close to the diagnosis, the growth curve showed an upward pattern; the proportion of PSN and SN rose as the main density types; and the appearance of poor morphological signs of nodules increased. When a persistent lung nodule starts to show a malignant change, a further diagnostic workup is warranted.

## Introduction

Lung cancer is the world’s leading cause of cancer death, and 75% of patients are diagnosed with advanced lung cancer (1). Early identification and treatment of lung cancer can help improve patients’ prognosis (1, 2). Low-dose chest CT is the current imaging method for lung cancer screening (2, 3). However, the evaluation of lung nodules by imaging examination is a complex and multifactor process, including the nodule position, size, density, morphological signs, and growth rate and even the specific conditions during scanning (4). Although some chest CT signs that are useful for identifying early lung nodules have been accumulated (5–8), there are still many unknowns about the biological characteristics and imaging signs of malignant nodules, which often leads to overdiagnosis and overtreated treatment (9, 10). Observing the natural growth behavior of lung nodules in the human body over a period is of great significance for distinguishing benign and malignant nodules in the early stage, understanding the biological characteristics of malignant nodules, and formulating lung nodule follow-up and management plans.

Currently, there are several models of lung cancer growth, including the exponential growth model and Gompertzian growth model. In 1956, Collins et al. (11) proposed an exponential growth model, which describes that the doubling time does not vary over the entire period of tumor existence. Mets et al. (12) observe 60 untreated solid and subsolid lung nodules with at least 3 follow-up images before diagnosis which supported lung nodule tumor growth as an exponential model. The Gompertzian growth model is a variation of the exponential growth model, which assumed that the tumor grows exponentially in the initial form, but the growth rate slows down as the tumor volume increases (13).

In this study, we collected 55 early lung nodules in 53 cases with pathologically confirmed results since 2003. These cases underwent continuous and regular (1-year interval) chest CT physical examinations. The size, density, and morphological sign changes of lung nodules were analyzed retrospectively, and the growth characteristics of malignant nodules explored, in order to understand the imaging characteristics of early lung cancer nodules.

## Materials and Methods

The first author (QD) had full access to and controlled all data in the study without any conflict of interest. This retrospective study was approved by the Institutional Review Board of Zhongshan People’s Hospital, and the requirement for written informed consent was waived. The population of this study has not been reported before.

### Study Population

**Figure 1:**
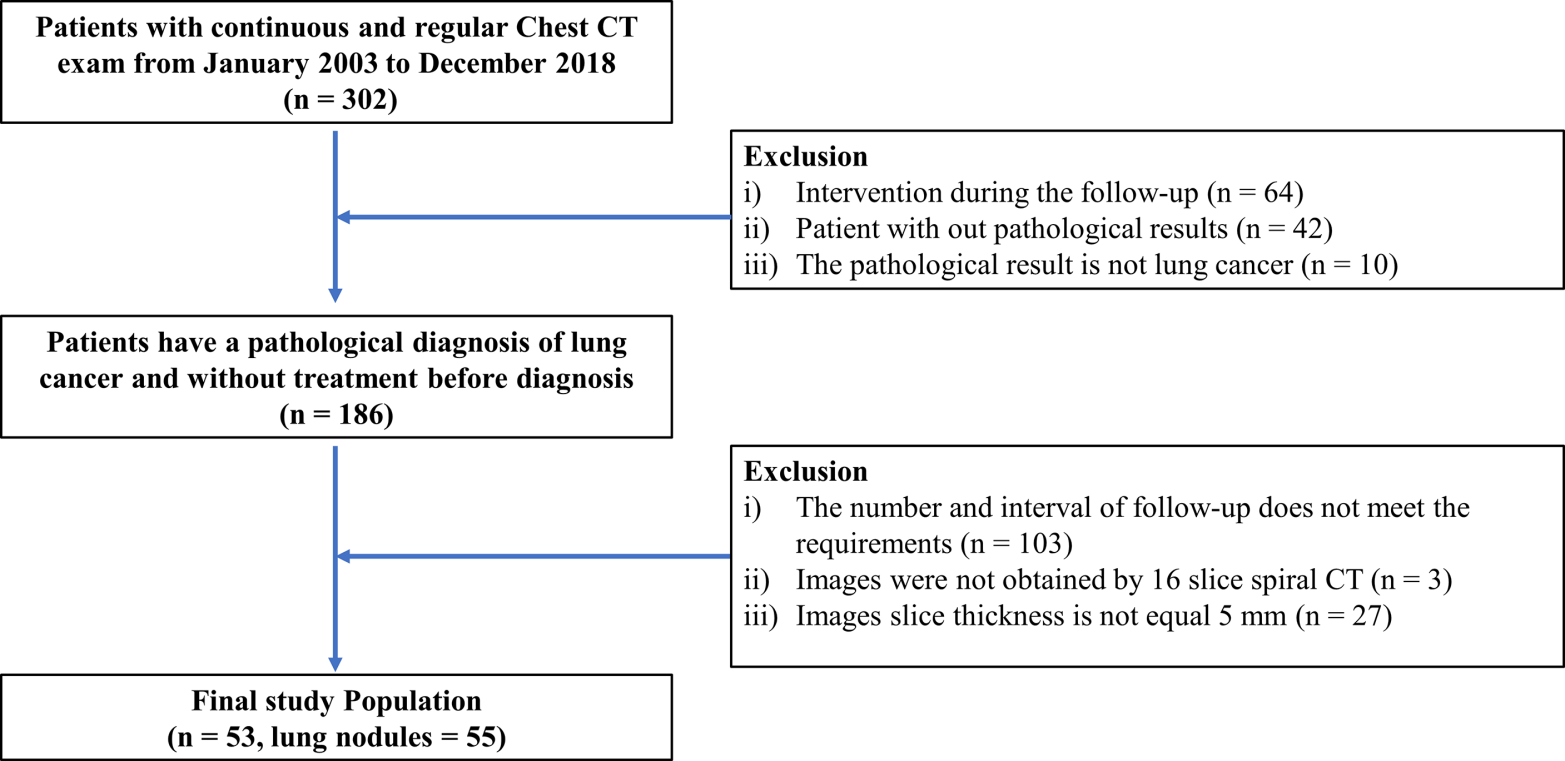
Flowchart for study enrollment.

Two radiologists (QD and XW, 3 and 2 years of experience in chest radiology, respectively) retrieved the physical examination database in our hospital and searched patients who underwent continuous and regular chest CT examinations and had pathologically confirmed lung cancer between January 2003 to December 2018. The inclusion criteria were as follows: a) no nodules were treated during observation; b) nodules were eventually diagnosed as lung cancer by pathology; c) patient had more than 3 chest CT follow-ups, with a follow-up interval of about 1 year; and d) all follow-up images were obtained from 16 slice spiral CTs, with a slice thickness of 5 mm. The flowchart for the study enrollment is shown in **Figure 1**. A total of 53 cases of early lung cancer with 55 nodules were collected.

### CT Examination

All the images were obtained by a 16-slice CT scanner (Philips Ingenuity Flex 16 or Siemens SOMATOM Emotion 16). The patient is reminded to hold their breath during the scan. The scanning parameters were as follows: tube voltage, 100 to 120 kV; tube current, 40 to 50 mAs; detector combination, 16 × 1.2 mm/16 × 0.6 mm; pitch, 1.0 to 1.3; scanning time, 13 to 20s. All CT image data were reconstructed with a slice thickness of 5 mm by a standard reconstruction algorithm.

### Demographic and Pathologic Data

We searched the electronic medical records of the study population and recorded the following: age, gender, follow-up years, lesion location, and pathology result. For cases prior to 2011, the pathological sections were reexamined by an experienced pathologist. The determination of pathological types in all patients is based on the lung tumor classification standard (released in 2011) (14).

### Radiologic Evaluation and Reverse Time Analysis

**Figure 2:**
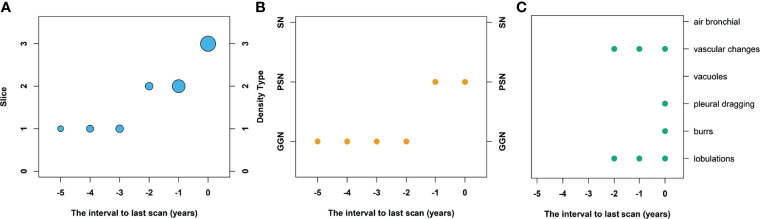
Reverse time map of the size, density, and morphological signs. **(A)** The reverse time-size bubble plot; **(B)** reverse time-density type scatter plot; **(C)** reverse time-morphological signs scatter plot from baseline scan to last scan.

The CT images were reviewed and recorded independently by two radiologists (QD and BT, 3 and 29 years of experience in chest radiology, respectively). Both were blinded to the follow-up phase, radiological report, and pathological findings of patients. When there was a discrepancy between the two radiologists, the final opinions are obtained after consultation between the two. The evaluation of the CT images focuses on the size, density, and morphological signs of the nodules. 1) The average diameter of the maximum slices of the nodule was used to represent size in an x–y direction, and the involved number of slices covered by the nodule was treated as size in the z-direction. A reverse time-size bubble plot was drawn with time as horizontal axis (unit in years), the size in z direction as vertical axis (unit in 5 mm), and the bubble size representing the size in the x–y direction (**Figure 2A**). 2) According to the views of the American Thoracic Association and the Japanese Clinical Oncology Group (15, 16), we calculated the ratio between the maximum diameter of the parenchymal component in nodule and the maximum diameter of the nodule under a CT lung window [-600 HU, 1,000 HU]. The parenchymal component refers to the part of the nodule with no recognizable blood vessels and bronchial structure. The density type of the nodule is classified as ground-glass nodule (GGN, ratio = 0, i.e., nodules that do not contain the parenchymal component), solid nodule (SN, ratio ≥0.5), and part-solid nodule (PSN, 0 < ratio < 0.5). A reverse time-density type scatter plot was made with time as horizontal axis (unit in years) and density type as vertical axis (**Figure 2B**). 3) Poor morphological signs of nodules: lobulation, burrs, pleural traction, vacuoles, vascular changes, and air bronchogram signs were accessed. A reverse time-morphological sign scatter plot was made with time as horizontal axis (unit in years) and poor morphological sign as vertical axis (**Figure 2C**). The presence, size, density, and morphological signs of the nodule at baseline (the first chest CT scan), follow-ups, and last (the CT scan obtained at the time of diagnosis of lung cancer) CT scan were recorded.

### Statistical Analysis

Continuous variables are presented as the mean and standard deviation (SD), and categorical variables are presented as numbers and percentages. Differences between groups were analyzed using the chi-square test or Fisher’s exact test for categorical variables and Student’s t-test for continuous variables. All statistical analyses were performed using SPSS statistics software (version 22.0, IBM SPSS Inc., Chicago, IL, USA). A two-sided p value of less than 0.05 was considered statistically significant.

## Results

### Clinical and Pathological Characteristics

A total of 55 lung nodules in 53 patients meeting the selection criteria were collected. The clinical and pathological characteristics are listed in **Table 1**. Of these, 62.3% (33/53) were men and 37.7% (20/53) were women. The average age of the patients at the time of baseline scans was 58.40 ± 11.43 years, and the average age of the patients at the time of diagnosis is 64.36 ± 11.43 years. The average number of follow-ups in this series was 6.52 ± 2.82 times, and the average follow-up period was 5.96 ± 2.68 years. There were 43.6% (24/55) of lung nodules located in the right upper lobe and 89.1% (49/55) of patients with lung adenocarcinoma.

**Table 1:** Clinical and pathological characteristics of selected patients.

Characteristics	Statistics
Gender	
Male	33 (62.3)
Female	20 (37.7)
Age at baseline scans	58.40 ± 11.43
Follow-up years	5.96 ± 2.68
Age at last scan	64.36 ± 11.43
Location	
Right upper lobe	24 (43.6)
Right middle lobe	3 (5.5)
Right lower lobe	6 (10.9)
Left upper lobe	16 (29.1)
Left lower lobe	6 (10.9)
Pathology	
Adenocarcinoma	49 (89.1)
Non-adenocarcinoma	6 (10.9)

Continuous data are reported as the mean and standard deviation, and categorical data as number (percentage).

### Observation of Nodule Size Changes Along the Follow-Up

**Figure 3:**
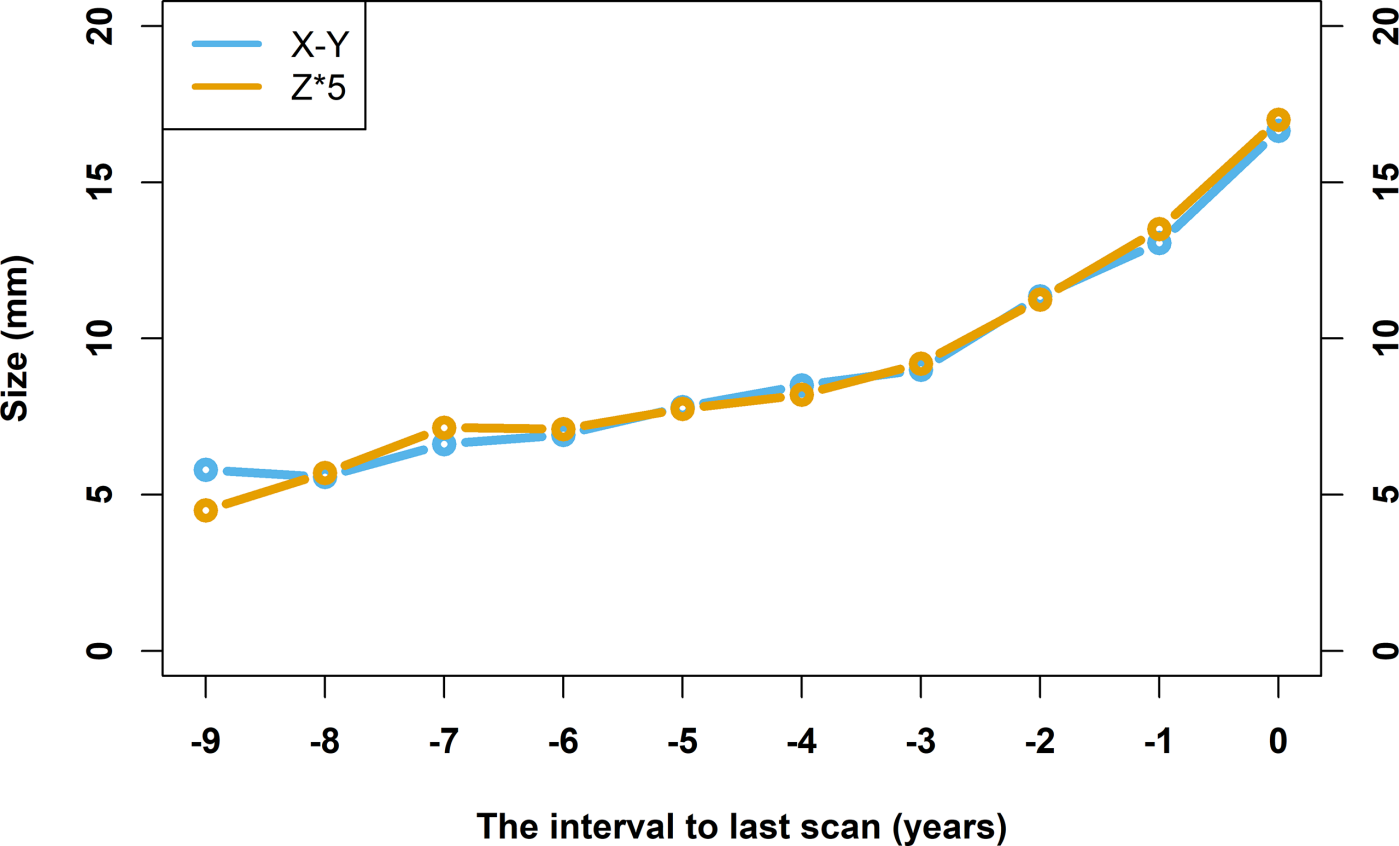
Line chart of nodule size changes over time. The horizontal axis takes the time of confirmed diagnosis as zero, and the rest of the time is defined relative to the time of confirmed diagnosis. The vertical axis indicates the size of the lesions, unit in mm. The line in yellow represents the average diameter of the largest slices, and the line in blue represents the product of the number of involved slices that lesions appeared in and the thickness of the slice (5 mm).

**Figure 4:**
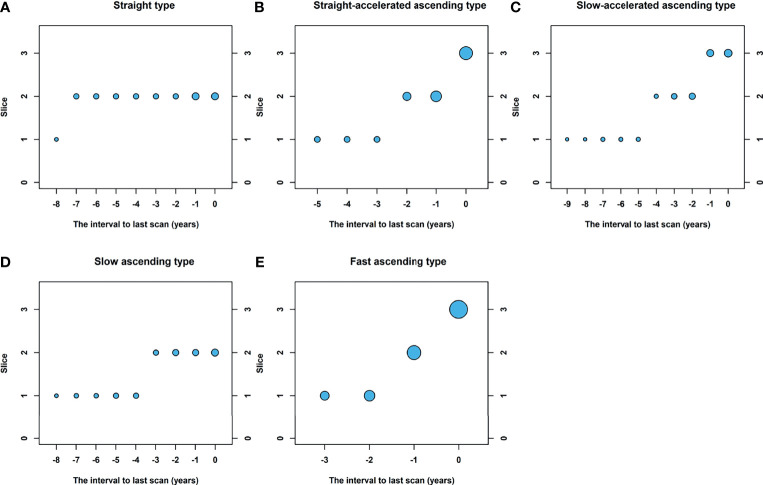
Schematic of five different types of growth curves. Based on changes in nodule size, five growth curves are summarized: **(A)** straight type, **(B)** straight-accelerated ascending type, **(C)** slow-accelerated ascending type, **(D)** slow ascending type, **(E)** fast ascending type.

In the baseline scan, 74.5% (41/55) of nodules were present and 25.5% (14/55) were not present. Among the 41 lesions, the average diameter of the largest slice is 6.83 ± 2.92 mm. At the time of diagnosis, the maximum slice of the nodules had an average diameter of 16.65 ± 7.34 mm. The involved slices in the baseline scan and last scan were 1.29 ± 0.46 and 3.40 ± 1.30 slices, respectively. **Figure 3** shows a line chart of changes in the size of the lung nodules. As you can see, with 4 years as the boundary, the line changes from a flat upward trend to a rapid upward trend. Based on changes in nodule size, five growth curves are summarized: straight type, straight-accelerated ascending type, slow-accelerated ascending type, slow ascending type, and fast ascending type (17). **Figure 4** is a schematic of five different types of growth curves. The follow-up time for the nodules of 5 different types of growth curves is shown in **Table 2**. The average follow-up time of the fast-rising type is 4.00 ± 1.76 years, and the average follow-up time of other types is greater than 4 years. Differences in follow-up times between different growth types are statistically significant (p < 0.001). There are 3 nodules that fluctuate in size at individual time intervals, and the lesions become smaller.

**Table 2:** Different types of growth curves and corresponding follow-up times.

Types of growth curve	Frequency (percentage)	Follow-up time (year)
Straight type	3 (5.5)	7.67 ± 0.58
Straight-accelerated ascending type	12 (21.8)	6.33 ± 2.15
Slow-accelerated ascending type	13 (23.6)	7.92 ± 3.23
Slow ascending type	15 (27.3)	5.20 ± 2.11
Fast ascending type	12 (21.8)	4.00 ± 1.76

Follow-up time as the mean and standard deviation, and frequency as number (percentage).

### Observation of Nodule Density Changes Along the Follow-Up

**Figure 5:**
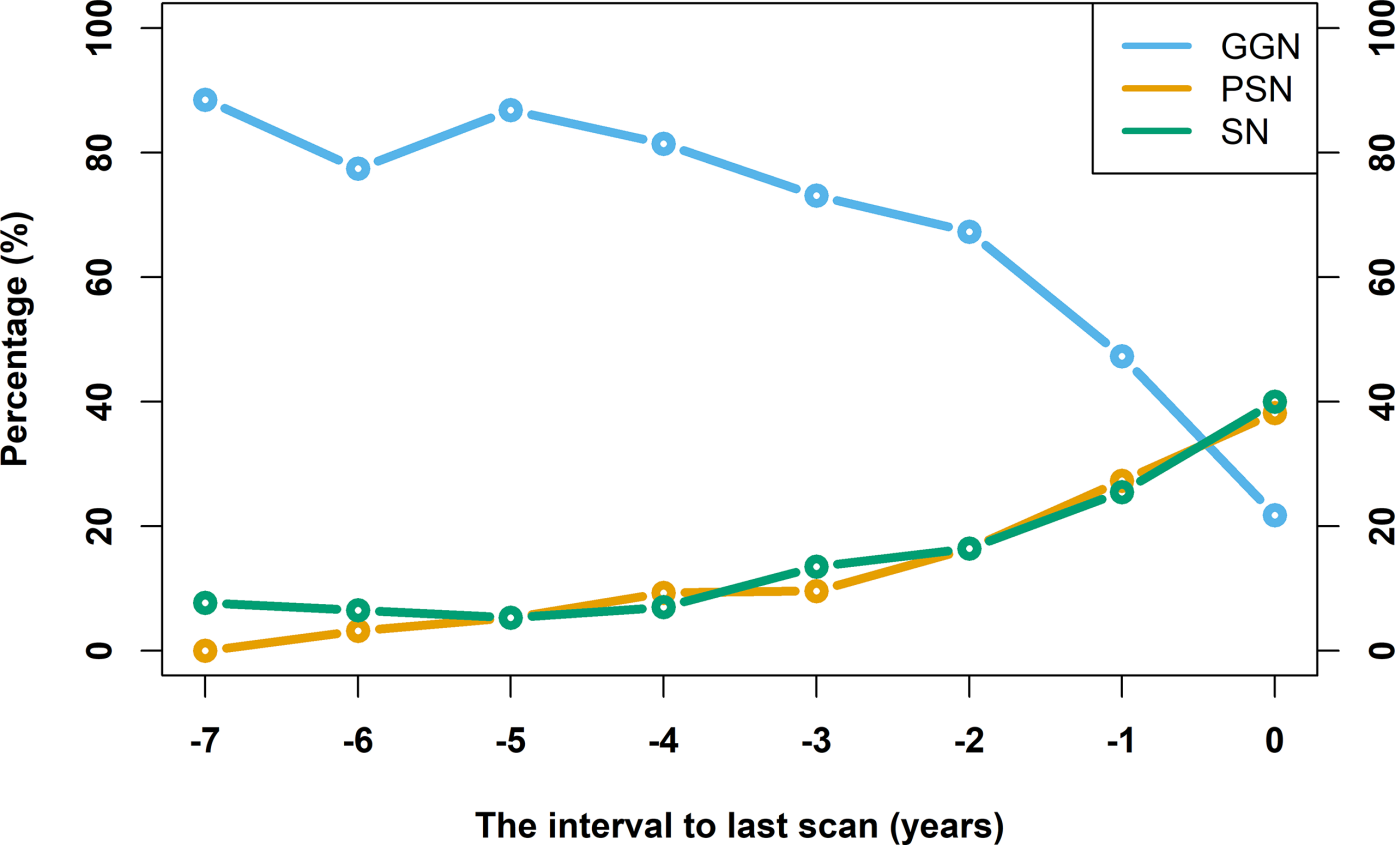
Line chart of nodule density type percentage changes over time. The horizontal axis takes the time of confirmed diagnosis as zero, and the rest of the time is defined relative to the time of confirmed diagnosis. The vertical axis indicates the percentage of density type.

Among 41 existing nodules in the baseline scan, there were 90.2% (37/41) GGN and 9.8% (4/41) SN. In the last scan, there were 21.8% (12/55) GGN, 38.2% (21/55), and 40.0% (22/55) SN. **Figure 5** shows a line chart of changes in the density type percentage of the lung nodules. It is shown in the figure that as time goes by, the percentage of GGN decreases, while the percentages of PSN and SN increase. GGN accounted for more than 80% beyond 4 years from the last scan (time of diagnosis), with fluctuations within 10%. Within 4 years from the last scan, the proportion of GGN dropped from about 80% to about 20%, and the closer the last scan, the greater the decline. PSN and SN accounted for a low level of less than 10% beyond 4 years from the last scan, and the proportions rose to 40% and 30.9% respectively within 4 years from the last scan. The density type conversion is shown in **Table 3**. There were 5 lesions that first appeared as SN, 4 of which were SN on baseline scan. The follow-up time of these nodules is relatively short.

**Table 3:** The density type conversion during follow-up times.

Density type conversion	Frequency (percentage %)
GGN	10 (18.2)
GGN→PSN	17 (30.9)
GGN→PSN→SN	10 (18.2)
SN	4 (7.3)
None→GGN	2 (3.6)
None→GGN→PSN	4 (7.3)
None→GGN→PSN→SN	5 (9.1)
None→GGN→SN	2 (3.6)
None→SN	1 (1.8)
Total	55 (100)

GGN, ground-glass nodule; SN, solid nodule; PSN, part-solid nodule.

Frequency as number (percentage).

### Observation of Nodule Morphological Signs With Changes During Follow-Up

**Figure 6:**
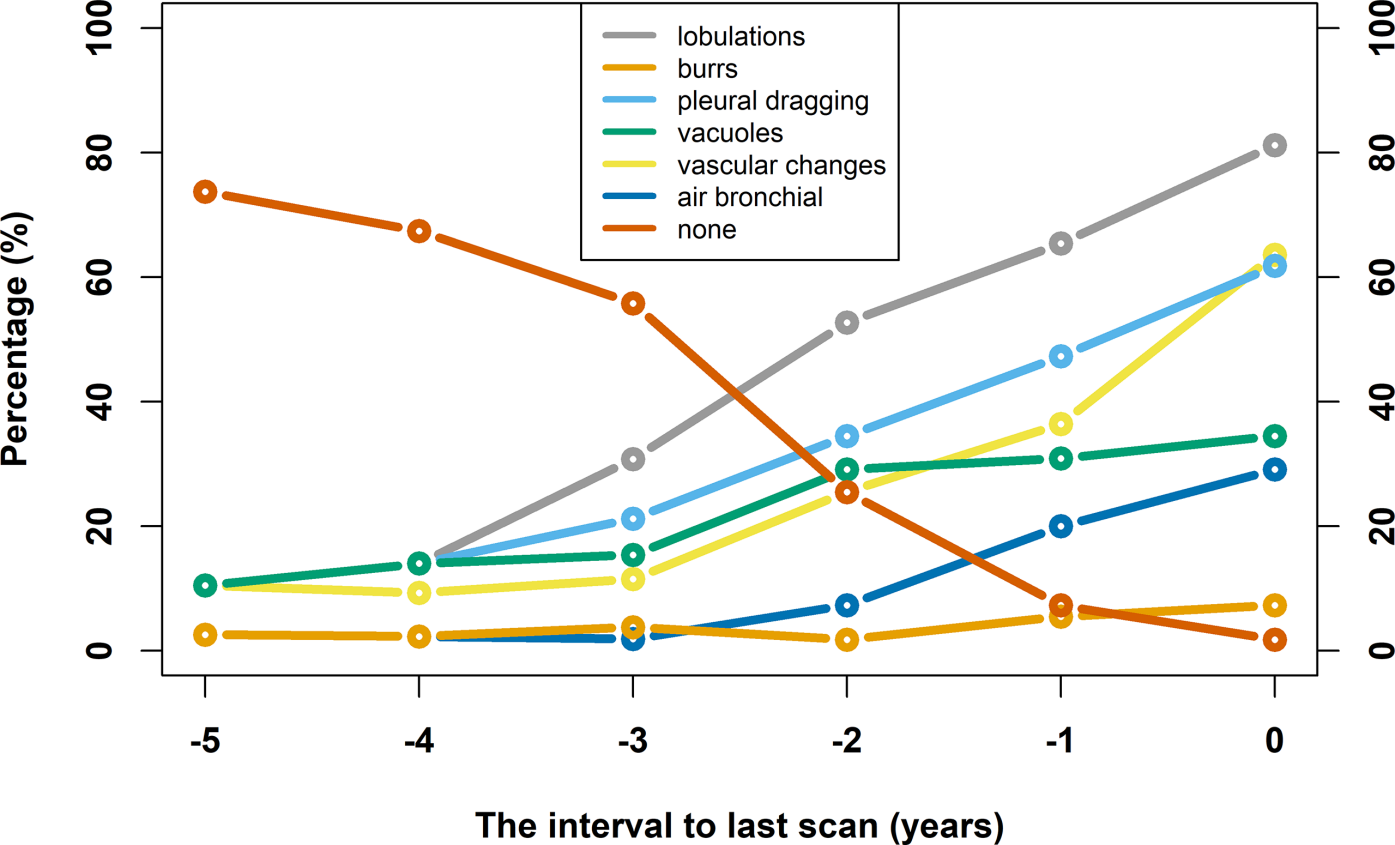
Line chart of nodule poor morphological signs changes over time. The horizontal axis takes the time of confirmed diagnosis as zero, and the rest of the time is defined relative to the time of confirmed diagnosis. The vertical axis indicates the percentage of poor morphological signs.

At baseline scan, 48 nodules had benign morphological signs (87.3%) and 7 nodules had poor morphological signs (12.7%). During the follow-up process, the frequency statistics of the earliest poor morphological signs of nodules are as follows: none (1/55, 1.8%), 31 marginal lobulation signs (31/55, 56.4%), 8 burr signs (8/55, 14.5%), 17 pleural dragging signs (17/55, 30.9%), 3 vacuole signs (3/55, 5.5%), 19 vascular change signs (19/55, 34.5%), and 0 air bronchial signs. The frequency statistics of poor morphological signs of nodules in the last scan are as follows: none (1/55, 1.8%), 45 marginal lobulation signs (45/55, 81.2%), 35 burr signs (35/55, 63.6%), 34 pleural dragging signs (34/55, 61.8%), 16 vacuole signs (16/55, 29.1%), 19 vascular change signs (19/55, 34.5%), and 4 air bronchial signs (4/55, 7.3%). **Figure 6** shows a line chart of changes in poor morphological signs of the lung nodules. The occurrence rate of poor signs increases along with time. In addition, the follow-up time is shorter for those lesions that show poor signs on the baseline scan.

### Case Image

**Figure 7:**
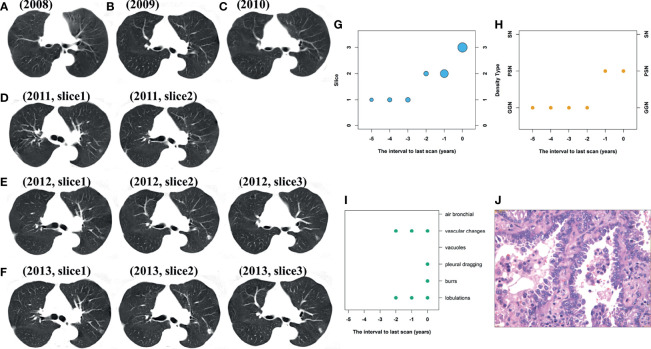
Female patients, 57 years old at baseline scan; the pathology result is invasive adenocarcinoma. **(A–F)** Are the annual screening CT images from 2008 to 2013; **(G–J)** are the changes in size, density, morphological signs, and pathology, respectively. From the baseline scan to the last scan, the lesion changed from 1 slice to 3 slices, the density changed from GGN to PSN. There are no poor morphological signs at the baseline and start to appear in follow-up scan.

**Figure 7** shows a 57-year-old female with a 6-year follow-up, including the changes in the annual screening image (A–F), size (G), density (H), morphological signs (I), and pathology images (J). In the 2 years before the last scan, the size of lung nodules in the x–y direction and z direction increased, and malignant characteristics began to appear. In the 1 year before the last scan, lung nodules changed from GGN to PSN.

## Discussion

This study collected 55 early lung nodules in 53 cases confirmed by pathology since 2003. These cases have undergone continuous and regular chest CT examinations (once a year). The size, density, and morphological sign changes of lung nodules along time were retrospectively observed and analyzed, and the growth characteristics of malignant nodules on the time axis are explored, in order to understand the comprehensive imaging signs of early lung cancer nodules. The dynamic observation results show that the malignant nodules have the characteristics of continuous existence and continuous growth. At the follow-up time furthest away from the confirmed diagnosis, the nodule density is dominated by GGN, most of which is without poor morphological signs, and the growth curve is flat; at the follow-up time closest to the confirmed diagnosis, the proportion of GGN decreases, and the proportion of PSN and SN increased, the appearance and increase of poor morphological signs of nodules are observed, and the growth curve presents an upward pattern.

The poor morphological features considered in this article include lobulation, burrs, pleural traction, vacuoles, vascular changes, and air bronchogram signs. These are clinically common signs for malignant nodules (18). The pathological basis of signs of lobulation, burrs, and pleural traction is the anisotropic growth of the nodules, the spread of tumor cells around the alveolar septum, and the involvement of tumor cells in the pleura. These signs have important value in judging benign and malignant nodules (19). The vacuoles and air bronchogram signs are due to the adherent growth of tumor cells, resulting in the presence of undamaged bronchioles and alveolars in the nodules, while tumor cells and adjacent fibrous tissues proliferate, so that the adjacent bronchus is stretched and expanded (20). Fibrosis in the nodules was the main reason for the morphological changes of the surrounding blood vessels (vascular changes). The collapse and fibrosis of the alveolar wall in the nodules dragged the surrounding blood vessels and changed the original direction or/and morphology of these blood vessels. When the tumor cells spread along the vascular bundle or infiltrated and grew along the interlobular septum, local blood vessels could be displaced, distorted, thickened, or cancerous embolus formed (21).

There are 3 nodules that fluctuate in size at individual time intervals, and the lesions become smaller. In Wang (22) and other studies, the growth curve of 2 cases of subsolid lung nodules showed a downward trend and then an upward trend. It is speculated that it may be due to changes of the lesion itself (such as absorption of inflammation) or be affected by breathing or observation errors. Literature suggests that growth of lung nodules can be non-exponential or exponential (22–24). Due to the short follow-up time and the randomness of the follow-up time interval, these literatures are speculated to only show the morphological characteristics of some segments of the growth curve of early lung cancer nodules.

The tumors that are manifested as GGN may be pre-invasive lesions (AAH, AIS) and invasive adenocarcinoma (25). It is reported that about 20% of lung adenocarcinomas appear as pure GGN nodules on CT, and the malignant tumor rate of pure GGN is 18% (26). It takes about 3.5 years for the infiltrating component to appear in GGN (27), and the doubling time can be up to 9 years (28). A total of 12 nodules in this group continued to be GGN from the first scan to the last scan (12/55, 21.8%). For the GGNs that exist for a long-time during follow-up, it is recommended to continue follow-up to rule out malignant growth. We found that the nodules start to show malignant change 4 years prior to diagnosis. Pathological examination is recommended for early diagnosis of lung cancer when nodules start to show a malignant change.

The advantages of this paper are as follows: Firstly, this series describes changes found in malignant lung nodules by a sequential CT chest exam over follow-up periods of up to 6 years in a single institution. Secondly, a reverse time analysis method is proposed. It provides a new analytical perspective on the observation of lung nodules. This method may be beneficial for early screening of lung cancer.

This article also has some limitations. Firstly, the number of cases included in this article is small. The main reason is the limited long-term follow-up data and strict inclusion criteria. The findings of this article need to be validated on a larger dataset. Secondly, the thickness of all CT images analyzed is 5 mm since the hardware limitations of the early follow-up stage. The volume calculations may not be accurate for small nodules. Therefore, the average diameter of the maximum slices of the nodule and the involved number of slices covered by the nodule were analyzed in this article and observed by drawing a reverse line chart. Thirdly, this article may show selection bias which tends to include smaller and slow-growing nodules. In future research, more detailed research is needed in a larger and multicenter data with thinner thickness.

## Conclusion

In summary, this article observed and analyzed the changes in the size, density, and morphology of the nodules in CT images before the pathological confirmation of early lung cancer. The analysis of the natural growth behavior of lung nodules may be helpful for distinguishing benign and malignant nodules, understanding the biological characteristics of malignant nodules, and making follow-up and management plans for lung nodules. It is suggested that routine follow-up of pulmonary nodules should be carried out. When the nodules start to show a malignant change, a further diagnostic workup is warranted.

## Data Availability Statement

The data analyzed in this study is subject to the following licenses/restrictions: Please contact the author by email. Requests to access these datasets should be directed to BT, zstangbh@sina.com.

## Ethics Statement

The studies involving human participants were reviewed and approved by the Ethics Committee of Zhongshan People’s Hospital. Written informed consent for participation was not required for this study in accordance with the national legislation and the institutional requirements.

## Author Contributions

QD: conceptualization, methodology, visualization, writing—original draft. JP: methodology, investigation, data curation. XW: investigation, data curation. MFJ: methodology, formal analysis. YL: formal analysis, visualization. BT: conceptualization, writing—review and editing, visualization, project administration. All authors contributed to the article and approved the submitted version.

## Conflict of Interest

Author YL was employed by company GE Healthcare Pharmaceutical Diagnostics.

The remaining authors declare that the research was conducted in the absence of any commercial or financial relationships that could be construed as a potential conflict of interest.

## Publisher’s Note

All claims expressed in this article are solely those of the authors and do not necessarily represent those of their affiliated organizations, or those of the publisher, the editors and the reviewers. Any product that may be evaluated in this article, or claim that may be made by its manufacturer, is not guaranteed or endorsed by the publisher.

## Abbreviations

GGN, ground-glass nodule; SN, solid nodule; PSN, part-solid nodule.
